# Molecular characterization of leaf spot fungi using internal transcribed spacer (ITS) based phylogenetic inference

**DOI:** 10.6026/97320630015046

**Published:** 2019-02-03

**Authors:** Bose Chitrakani, Senthuran Sureshkumar, Pandy Rajapriya, Mohan Pandi

**Affiliations:** 1 Department of Molecular Microbiology,School of Biotechnology,Madurai Kamaraj University,Madurai- 625 021,Tamil Nadu,India; 2 Department of zoology, M.S.S. Wakf Board college,Madurai - 625 020, Tamil Nadu, India

**Keywords:** Leaf spot fungi, ITS, secondary structure, phylogenetic tree

## Abstract

The plant pathogenic leaf spot fungi cause loss in crop yield. Fungi and other pathogens such as a virus, bacteria, cause leaf spot diseases and nematodes play a secondary role. Therefore, it is of interest to study internal transcribed spacer (ITS) sequence from the plant pathogenic fungi. Hence, we collected nineteen different isolates at Madurai Kamaraj University, Tamilnadu, India for this study. We report nineteen positive isolates identified with species-level characterization using ITS sequence supported with a phylogenetic tree and corresponding secondary structure analysis.

## Background

Plants are an excellent source for the innovation of new products with medicinal importance in drug development. Today, lots of different chemicals derived from plant sources, which are presently used in more countries in the world. The secondary metabolites are economically very important as drugs, fragrances, pigments, flavors, food sources, and pesticides. Many of the drugs sold in the present day were simply synthetic modifications or copies of the naturally obtained substances. The growing commercial significance of secondary metabolites has in current years resulted in a good concern in secondary metabolism [1]. All the Fungi are rich sources of thousands of secondary metabolites. Only a few reports available about Leaf spot fungi and their identification at the species level. The probable reason is the identification of a single organism which in teleomorph and anamorph variation is not for all time similar [2-3]. The nuclear rDNA internal transcribed spacer region has confirmed as one of the more frequently utilized regions for phylogenetic analysis than other gene markers such as Cytochrome oxidase c (cox), Beta-tubulin and others [4-5]. The ITS region is composed of ITS1/ITS2 intergenic sequences with well conserved 5.8 rRNA in between. However, still less trouble in naming down the species level due to dissimilar errors. So ITS2 became fast evolving and it is a small non-coding region located inside the nuclear ribosomal DNA cluster. ITS2 sequence variability is thought to be suitable to differentiate species and for phylogenetic reconstructions, which can be further enhanced if structural information is considered. The double-edged tool contains <200 base pairs used for comparing eukaryotic evolutionary correlation [6]. Molecular characterization has huge potential to additional kind of fungal biodiversity and ecological divisions. The ITS region is the universally sequenced genetic marker for fungal identification. Nowadays, the ITS has commonly used the sequence for many studies and fourteen thousand fully known fungal species available in the public sequence databases [7]. The sequence information that can be found from the secondary structure of the nuclear ribosomal internal transcribed spacer 2 (ITS2) is essential, and thus far many studies develop this information erratically or improperly. They initiate a remedy in the form of a flowchart where we specify the steps concerned in estimating structure-based phylogenetic trees from ITS2 data. The channel explains consists of the ITS2 Database, 4SALE, the CBC Analyzer, and Prof DistS software. Based on these software implements; they express closely how to useITS2 sequence and secondary structure based information along with an ITS2 particular scoring matrix and an ITS2 specific additional model. As well as, compensatory base changes (CBCs) in ITS2 secondary structure pairs are known as a possible marker for individual species [8]. The present study was elaborated on molecular identification of leaf spot fungi based on ITS region and ITS2 secondary structures.

## Methodology

### Collection of Samples:

Infected plant leaf samples were collected from different sites of Madurai Kamaraj University (MKU), Madurai, Tamilnadu, India. The samples were sealed and shifted to the laboratory in a sterile polythene cover. The leaves were washed with running tap water in order to remove dust and debris. The diseased part of a leaf from each plant sample was cut ~1cm. The removed leaf segments were surface sterilized by sequential rinses in 70% ethanol for 5 sec, 4% sodium hypochlorite for 90 sec and in sterile distilled water for 10 sec [9]. The surface sterilized leaf parts were placed in Petri plates holding sterile tissue paper to remove extra moisture under sterile condition. After that, the surface sterilized leaf segment was embedded in Petri dishes containing solid potato dextrose agar medium, with 50 mg/L streptomycin sulfate added to avoid bacterial growth. Now, the plates were incubated at 25°C temperature with 12h light/ 12h dark cycle. The plant segments were monitored after a day for the growth of fungi. Hyphal tips emerged out the plated parts were instantly transferred into PDA slant and maintained at 4 °C [10].

### DNA extraction, amplification of ITS region and sequencing:

The Genomic DNA was extracted from the Leaf spot fungi using cenis et al. [11] method. The partial nucleotide internal transcribed spacer (ITS) rDNA region was amplified from the genomic DNA by using the polymerase chain reaction (PCR) [ITS1 forward primer (5'TCC-GTA-GGTGAA- CCT-GCG-G 3'); ITS4 reverse primer (5'TCC-TCCGCT- TAT-TGA-TAT-GC3')] [12]. The PCR amplification was performed in a Bio-RAD instrument with a total 25 µl reaction comprised of 20 ng of genomic DNA template, 10X buffer with 25mM MgCl2, 10mM dNTP's, 2U of Taq DNA polymerase and 10 pmol of each primer (Sigma -Aldrich). The following PCR reaction conditions were used: 4 min at 94°C for denaturation, 30 cycles each of 30 sec at 94°C for denaturation, 1min at 58.2°C for annealing, 2 min at 72°C for extension followed by the final extension at 72°C for 7 min. The amplified ITS region evaluated by 1% agarose gel electrophoresis with a 100bp marker obtained from Bangalore (Genei) and the amplified products were visualized by under a gel documentation system (Gel logic 2200 PRO). A non-template (negative control) was included in each run. Further, the amplicons were sequenced by Euro Fins Private Limited, Bangalore, India.

### Internal Transcribed Spacer 2 (ITS2) secondary structure prediction, alignment, Phylogenetic analysis:

ITS sequences of our fungal isolates and control sequences were used for phylogenetic analysis (Neighbour-joining method with 1000 bootstrap replications) using MEGA 5.1. Fungal ITS2 regions were extracted using fungal ITS extractor software. The selected secondary structures were downloaded in a Vienna file format from Mfold server [13-14]. ITS2 sequences and secondary structures were aligned using the software 4SALE V 1.7, and final alignment was exported to ProfDistS [15] for tree construction. The consensus structure of each genus was generated using 4SALE V 1.7 software[16].

## Results

**Figure 1:**
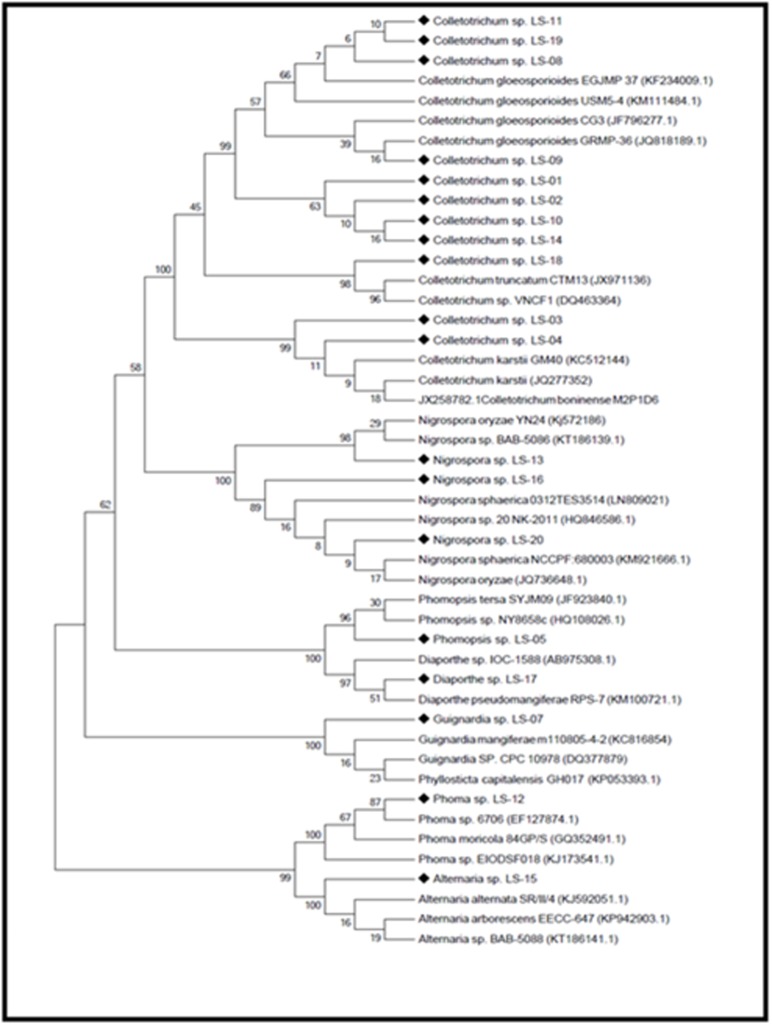
Phylogenetic analysis of ITS region derived from Neighbour- Joining method using MEGA 5.1 software. (Diamond shapes represent our isolates and remaining are reference sequences).

The present study is focused on exploring the leaf spot fungi from different medicinal plants. Overall 19 leaf spot fungi were isolated based on the culture morphology, color and mycelial growth patterns. 19 isolates were differentiated into six genera such as Colletotrichum sp., Diaporthe/Phomopsis sp., Guignardia sp., Phoma sp., Nigrospora sp., Alternaria sp, (Table 1). The 19 organisms were taken for molecular identification using ITS region. Based on the PCR analysis the range of 500 to the 600bp length of ITS regions was observed in 1% Agarose Gel Electrophoresis. The purified amplicons were sequenced using both forward primer and reverse primer. Both sequences were merged using EMBOSS Merger and full-length ITS sequences were intended for BLAST analysis. Query sequences named after the similarity with the maximum score and those query coverage sequences were identified and the phylogenetic tree was constructed (Figure 1). Further, all the nineteen isolates were subjected to ITS2 secondary structure-based analysis. Fungal ITS extractor programme used to separate the ITS2 region and predict secondary structure using Mfoldserver. The Vienna files were downloaded and analyzed the secondary structures information. The minimum free energy of secondary structures was observed and it's ranged from -82.82 to-51.79 kcal/mol (Table 2).

## Nucleotide information of Query and Reference sequences

**Figure 2:**
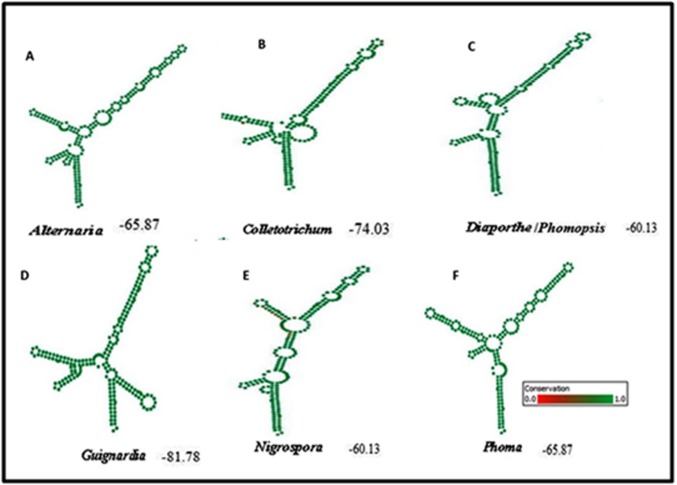
Consensus ITS2 RNA secondary structure of Leaf spot fungi A) Alternaria sp., B) Colletotrichum sp., C) Diaporthe sp/Phomopsis sp., D) Guignardia sp., E) Nigrospora sp., and F) Phoma sp.

Forty sequences were subjected to nucleotide analysis: nineteen was our query sequence and twenty-one for reference sequences collected from the NCBI database. The length of the ITS2 sequences ranged from 187-203nt, and GC content detected in the percentage 48.92 to 57.92 (Table 2). In addition, all the sequences were used to model consensus structure for each genus (Figure 2). The Guignardia sp. shared the four-helix loop region as helixes second and third is recognizable. Alternaria sp., Colletotrichum sp., Diaporthe/Phomopsis sp., Nigrospora sp. Phoma sp. possessed three helixes and the third helix is the longest in the entire genus. This alignment was used to analyze the CBC within the genus and to construct the ITS2 Sequences secondary structure based phylogenetic tree.

**Figure 3:**
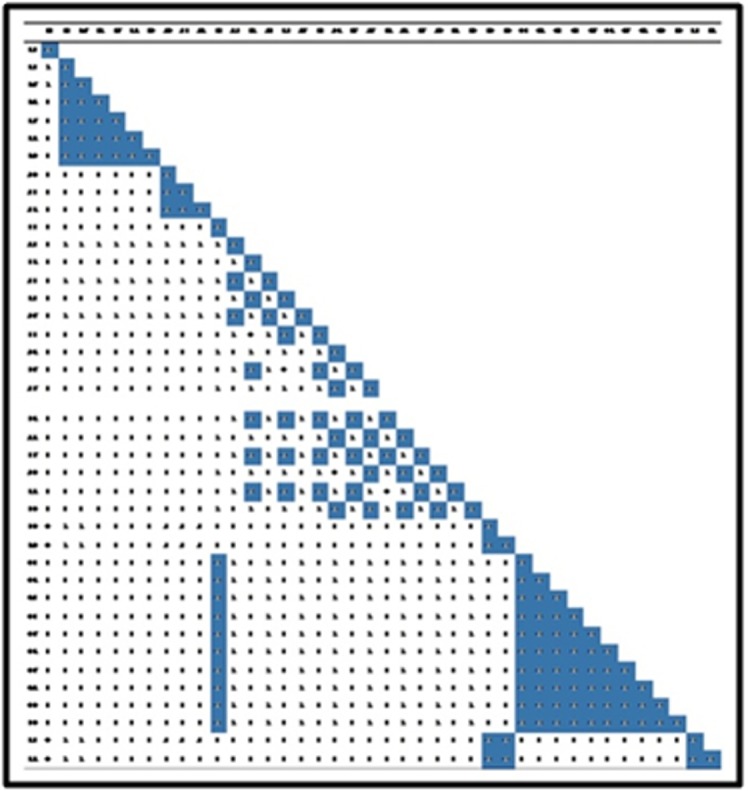
CBC information: Colletotrichum: 01-18, Phomopsis: 19-21, Diaporthe: 22 and 23, Nigrospora: 24-29, Guignardia: 30-32, Phoma: 33-35, Alternaria: 36-40.

**Figure 4:**
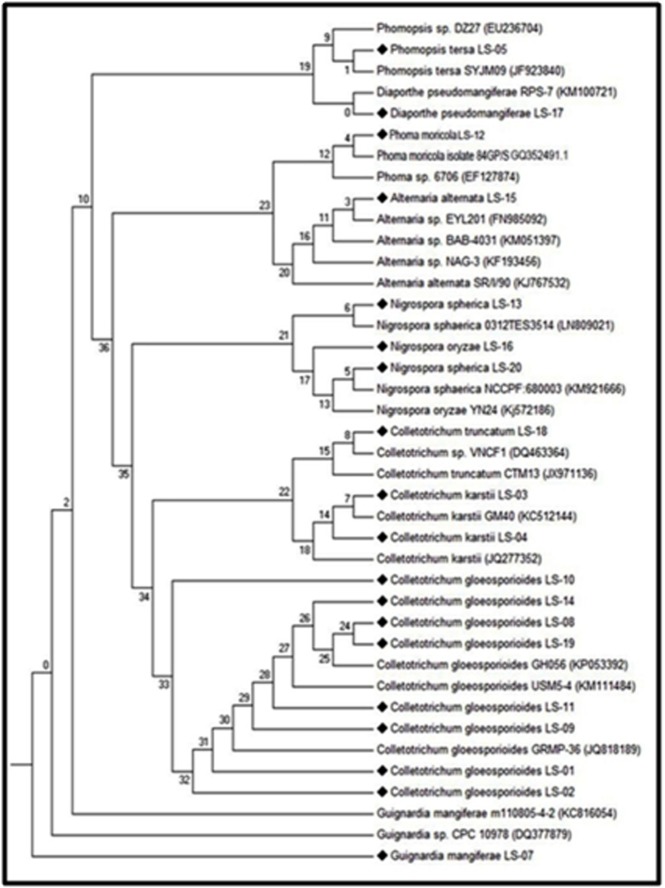
Neighbour-Joining tree obtained by ProfDistS, synchronously calculated on ITS2 sequence and secondary structure information, using an ITS2 specific General time reversible substitution model. Bootstrap values from 1000 pseudo-replicates (Diamond shapes represent our isolates and remaining are reference sequences).

In CBC analysis clearly distinguish the different genus i.e., Colletotrichum, Phoma, Phomopsis, Nigrospora, Alternaria, Guignardia and Diaporthe) and species (Colletotrichum gloeosporioides and Colletotrichum truncatum) with numerical values (greater than 0) (Figure 3). CBC value 1 or above among two isolates compared indicates the two isolates belong to two different species. While CBC value 0 denotes that the two isolates may belong to the same species. Some closely related species CBCs were not found in Colletotrichum sp., and Nigrospora., but insertion/deletion events (INDELS) were observed. The sequence secondary structure-based phylogenetic analysis also yielded wellresolved clades. The secondary structures of ITS2 were identified for eight queries and nineteen known isolates were obtained from Genbank database using Mfold programme with default conditions (Figure 4). ITS2 sequence secondary structure based phylogenetic analysis could be an important method for distinguishing closely related species. In Alternaria genus, secondary structures had been modelled for two queries, and four control sequences, the minimum free energy (MFE) was notified as -65.87. In the genus Phoma sp., has -60.13 and, 3 sequences of Nigrospora have - 60.13 MFE, whereas Diaporthe sequence is used which has -65.87 MFE correspondingly. Out of one sequence used in Guignardia have -81.78. Colletotrichum -74.03 and Phomopsis genus was found to be - 82.82 MFE. The above all 7 different genera were observed and their structure arrangement was represented. The 20 bp of the 5.8S and 28S rDNA which is present as flanking to the 5'-end and 3'- end of the ITS2 region apparently formed canonical bonds by each other. The phylotree was constructed for query and control ITS2 sequences using ProfDistS. Based on these analyses all the isolates belong to 5 order (Glomerellales, Diaporthales, Botryosphaeriales, Pleosphorales, and Trichosphaeriales), 6 Genus (Colletotrichum, Phoma, Diaporthe/Phomopsis, Nigrospora, Alternaria, and Guignardia) and 10 species (Colletotrichum gloeosporioides, Colletotrichum truncatum, Guignardia mangiferae, Nigrospora oryzae, Nigrospora spherica, Alternaria alternata, Phoma moricola, Phomopsis tersa, Colletotrichum karstii, and Diaporthe pseudomangiferae).

## Discussion:

In this study, nineteen leaf spot fungi were isolated and recognized down to species level using ITS sequence and ITS2 sequence secondary structure. The colonization efficiency of Leaf spot fungi influenced mainly by climatic conditions, plant age and sterilization methods etc. Based on the earlier reports, the diversity and identification of fungi down to species level was difficult without doing the molecular study [12,16]. In the present study, all the nineteen fungal isolates were subjected to molecular analysis using amplifying the ITS region and phylogenetic tree was constructed. The organisms were classified up to species level. The ITS (Internal transcribed spacer) is the one of the universal marker for fungal identification. The ITS region of the nuclear (rDNA) cistrons is one of the additional frequently utilized regions used for phylogenetic studies by the genus and species levels. It has long-established important for phylogenetic reconstruction of genus and species relations, by comparisons of primary sequence. Where as probable transcript secondary structure homology is regularly used to support alignment in comparisons of ribosomal gene sequences, such concern has rarely been applied to ITS primarily as secondary structures for its transcript was not available. Now, they identified the value of applying ITS2 region of RNA transcript secondary structure prediction to become better alignments, which following to that permits comparisons at however deeper levels of taxonomy. The evolutionarily conserved sub-portions of ITS2, it seems that needed for positioning of the multi molecular transcript processing technology, and provide material for distinguishing evolutionarily irregular events, Compensatory Base Changes (CBC) in the relatively conserved regions, that might be helpful in recognizing how unsystematic are the assignments of conventional taxonomic position of different groups in eukaryotes. This relatively short and easily sequenced region of DNA - ITS2 has so far to be fully subjugated in phylogenetics [4]. While significant variations in nucleotide sequences, the secondary structure of eukaryotic ITS2 has been shown to be highly conserved with four helices and some common motifs [18-19]. The molecular study of the genus, which is the most species-rich, Therefore, ITS2 RNA secondary structure investigation could be a valuable tool for distinguishing new species and completing systematic, morphological phylogenetic reconstruction of octo corals is very difficult ([20] and ITS2 secondary structure has more information than the usual primary sequence alignment [21-25,19]. Over 1,300 organisms were classified, different species of the same genus; CBCs were detected between 93% of the species. These results specify that a CBC in an ITS2 sequence-structure alignment offer strong evidence to distinguish species [19]. CBC can be an enough but not an essential criterion to differentiate between distinct species and the result of a CBC analysis may be used to estimate the minimal number of different species present in multiple alignments [26-27,8]. Correspondingly, in our results CBCs were found between different genus and species except between the genus of phomopsis, Diaporthe and because both are telomorphic and anamorphic stage respectively and among the Colletotrichum sp., (C.truncatum and C. karstii) and Nigrospora sp., (N.oryzae and N.sphaerica), but insertion/deletion events were observed. Based on these results conclude that ITS2 sequence-structure based phylogenetic analysis could be a valuable method for distinguishing closely related species.

## Conclusion:

We report the isolation and characterization of nineteen leaf spot fungal strains from different medicinal plant in Madurai Kamaraj University, Tamilnadu, India using molecular phylogenetic data, compensatory base changes (CBC) and secondary structures. The species identified through this study include Colletotrichum gloeosporioides, Colletotrichum karstii, Colletotrichum truncatum, Guignardia mangiferae, Nigrospora oryzae, Nigrospora sphaerica, Alternaria alternata, Phoma moricola, Phomopsis tersa and Diaporthe pseudomangiferae. Analysis shows that ITS2 sequence-structure based analysis could be a valuable way for distinguishing closely related species.

## Conflicts of Interest:

The authors declare no conflict of interest.

## Figures and Tables

**Table 1:** : Total number of fungal pathogens

S. No	Fungi	Number of isolates
1	Colletotrichum spp.	11
2	Diaporthe/Phomopsis sp.	2
3	Guignardia sp.	1
4	Phoma Sp.	1
5	Nigrospora spp.	3
6	Alternaria sp.	1

**Table 2:** Nucleotide information of query and reference sequences

S. No	Organism name	Genbank number	Total length	ITS2 region	A's	U/t's	G's	C's	GC%	MFE (37°)
1	Colletotrichum gloeosporioides coll914	MF671961	488	291-448	37	50	53	58	56.06	-74.03
2	Colletotrichum gloeosporioides BCKSKMP-2	MF685014	491	297-454	37	50	53	58	56.06	-74.03
3	Colletotrichum karstii BCKSKMP-3	MF685015	509	307-464	37	50	53	58	56.06	-67.33
4	Colletotrichum karstii BCKSKMP-4	MF685016	557	359-516	36	50	54	58	56.56	-73.43
5	Phompsis tersa BCKSKMP-8	MG049670	547	348-509	38	47	56	61	57.92	-82.82
6	Guignardia mangiferae BCKSKMP-15	MG265988	602	400-562	36	51	59	57	57.15	-81.78
7	Colletotrichum gloeosporioides BCKSKMP-7	MG242345	537	340-497	37	50	53	50	54.21	-74.03
8	Colletotrichum gloeosporioides BCKSKMP-9	MG049671	549	346-503	37	50	53	58	56.06	-74.03
9	Colletotrichum gloeosporioides BCKSKMP-12	MG242346	491	288-445	37	50	53	58	56.06	-74.03
10	Colletotrichum gloeosporioides BCKSKMP-11	MG242345	505	302-459	37	50	53	58	56.06	-74.03
11	Phoma moricola BCKSKMP-7	MF960791	511	311-467	39	59	48	51	50.25	-60.13
12	Nigrospor sphaerica BCKSKMP-5	MF960789	511	321-473	35	55	48	55	53.26	-60.13
13	Colletotrichum gloeosporioides BCKSKMP-10	MG242344	537	342-499	37	50	53	58	56.06	-74.03
14	Alternaria alternate BCKSKMP-17	MG265990	498	304-462	37	62	49	51	50.25	-65.87
15	Nigrospora oryzae BCKSKMP-6	MF960790	435	278-435	36	57	46	55	52.06	-64.66
16	Diaporthe pseudomangiferae BCKSKMP-16	MG265989	550	347-505	36	57	47	55	52.3	-65.87
17	Colletotrichum truncatum BCKSKMP-13	MG242347	557	345-501	40	46	55	58	56.78	-78.65
18	Colletotrichum gloeosporioides BCKSKMP-18	MG265991	517	313-470	35	57	53	52	53.29	-69.12
19	Nigrospora spherica BCKSKMP-14	MG265987	501	320-474	37	50	53	58	56.06	-74.03
20	Colletotrichum gloeosporioides GH056	KP053392	547	346-502	36	57	47	55	52.3	-65.85
21	Colletotrichum gloeosporioides USM5-4	KM111484	580	364-521	37	50	53	57	53.83	-74.09
22	Colletotrichum gloeosporioides GRMP-36	JQ818189	497	298-455	37	50	55	58	56.5	-74.03
23	Colletotrichum sp. VNCF1	DQ463364	522	354-522	37	50	53	58	55.5	-74.03
24	Colletotrichum truncatum strain CTM13	JX971136	610	392-548	33	56	49	51	52.91	-60.32
25	Colletotrichum karsti	JQ277352	523	319-548	37	54	54	52	54.63	-71.93
26	Colletotrichum karstii GM40	KC512144	563	360-517	36	50	54	58	56.56	-73.43
27	Phomopsis tersa SYJM09	JF923840	562	341-502	36	50	54	58	56.56	-73.43
28	Phomopsis sp. DZ27	EU236704	595	372-533	39	47	55	61	57.42	-76.32
29	Diaporthe pseudomangiferae RPS-7	KM100721	554	351-509	38	47	56	61	57.63	-82.82
30	Nigrospora sphaerica 0312TES35I4	LN809021	547	381-547	40	46	55	58	56.78	-78.65
31	Nigrospora oryzae YN24	KJ572186	538	337-491	34	54	44	55	52.14	-51.79
32	Nigrospora sphaerica NCCPF:680003	KM921666	585	372-526	36	57	47	55	54.3	-65.87
33	Guignardia sp. CPC 10978	DQ377879	1483	400-562	36	51	59	57	57.14	-81.78
34	Guignardia mangiferae M110805-4-2	KC816054	639	419-581	36	51	59	57	57.14	-81.78
35	Phoma moricola	GQ352491	545	330-486	39	59	48	51	50.25	-60.13
36	Phoma sp. 6706	EF127874	543	329-485	39	59	40	51	48.92	-60.13
37	Alternaria sp. EYL201	FN985092	530	323-481	37	62	49	51	50.25	-65.25
38	Alternaria sp.NAG-3	KF193456	528	311-469	37	62	49	51	50.25	-65.25
39	Alternaria sp.BAB-4031	KM051397	564	336-494	37	62	49	51	50.25	-65.25
40	Alternaria alternata SR/I/90	KJ767532	526	310-468	37	62	49	51	50.25	-65.25

## References

[1]  Safaraj MD (2012). J Pharm Bioallied 4.

[2]  Kuhls K (1997). Mycologia.

[3]  Rehner SA, Samuels GJ (1995). Can J Bot.

[4]  Coleman AW (2003). Trends Genet.

[5]  Villa-Carvajal M (2006). Querol, A Belloch, DNA gene Antonie van Leeuwenhoek.

[6]  Wolf M (2013). PLOS ONE.

[7]  Begerow D (2010). Appl microbiol biotechnol.

[8]  Schultz J, Wolf M (2009). Mol Phylo Evol.

[9]  Kumaran RS (2009). Biotechnology And Bioprocess Engineering.

[10]  Guo-Yin T (2013). Afr J Microbial Res.

[11]  Cenis JL (1992). NuclAciRes.

[12]  Sim J (2010). J Microbiol Biotechnol.

[13]  Zuker M (2003). Nucleic Acids Res.

[14]  Meyer IM, Miklos I (2007). PLoSComput Biol.

[15]  Friedrich J (2005). Bioinformatics.

[16]  Won H, Renner SS (2005). Mol Phylogenet Evol.

[17]  Joseph N (1999). Nucleic Acids Res.

[18]  Coleman AW (2007). Nucleic Acids Res.

[19]  Ahvenniemi P (2009). J Mol Evol.

[20]  Aguilar C, Sanchez JA (2004). Bull Marine Sci.

[21]  Aguilar C, Sanchez JA (2007). Mol Phylogenet Evol.

[22]  Coleman AW, Vacquier VD (2002). J Mol Evol.

[23]  Tippery NP, Les DH (2008). Mol Phylogenetic Evol.

[24]  Chen S (2010). PLoS ONE.

[25]  Buchheim MA (2012). Ann Bot.

[26]  Glass DJ (2013). Mol Phylogenet Evol.

[27]  GokulRaj K (2014). Bioinformation.

